# Editorial: Promoting and exploring the effectiveness of the psychological first aid approach

**DOI:** 10.3389/fpubh.2024.1475151

**Published:** 2024-08-20

**Authors:** Moshe U. Farchi, Alba Pérez-González, Susana Celeste Azzollini, Yori Gidron

**Affiliations:** ^1^Department of Social Work, Research Center for Innovation in Social Work, Tel-Hai College, Upper Galilee, Israel; ^2^Department of Psychology and Education Science, Open University of Catalonia, Barcelona, Spain; ^3^National Scientific and Technical Research Council (CONICET), Buenos Aires, Argentina; ^4^Department of Nursing, University of Haifa, Hifa, Israel

**Keywords:** psychological first aid, acute stress reaction, PTSD prevention, six Cs model, YaHaLom, sense of coherence

The necessity for Psychological First Aid (PFA) arises from the need to address acute stress reactions (ASRs) that individuals experience following potentially traumatic events (PTE). ASRs, characterized by anxiety, confusion, and helplessness, can severely impair a person's ability to function. Without timely intervention, ASR can escalate, leading to long-term psychological problems like post-traumatic stress disorder (PTSD) (1). Effective PFA aims to stabilize individuals immediately, reducing the risk of developing chronic mental health problems and aiding in the rapid return to normalcy.

Initially, interventions such as psychological debriefing were widely used. This model focused on encouraging emotional expression and recounting traumatic experiences shortly after the event. However, several meta-analyses found that debriefing is ineffective and sometimes increased the risk of PTSD (2).

Modern approaches to PFA emphasize immediate, cognitive-functional interventions designed to stabilize individuals and promote resilience. One such model is the Six Cs, developed by Farchi et al. (3), which focuses on Cognitive Communication, Challenge, Continuity, Control, and Commitment. This model aims to shift individuals from helplessness to active coping by engaging cognitive and behavioral processes to regulate emotional responses, thus reducing the impact of limbic system hyperactivity (3). Studies have shown that the Six Cs model effectively reduces anxiety and improves resilience and self-efficacy in both professionals and laypersons trained to respond to ASRs (4). The Israeli defense forces and other armies have adopted the Cs in the form of YaHaLOM, attesting to the applicability of the Cs in other contexts and cultures (5).

Studies were conducted to determine the effectiveness of PFA training based on the protocol developed by Farchi et al. (3). Additionally, were more effective in all cases, especially when the rescuers were trained (6). In fact, trained rescuers who were instructed to make decisions emotionally were as inefficient as untrained ones. Moreover, those with high openness to experience, high honesty, high attraction and low aversion to culturally diverse others, and low avoidant coping were the ones with the greatest ability to provide effective responses in PFA (7).

## Challenges that PFA approach must address

Research over the years reaffirms the importance of early intervention in offering basic support to people who have experienced a PTE (8). This support should focus on cognitive assessments, promote coping resources, and help reduce ASR by facilitating the return to normal functioning and regulating stress responses.

Professionals must approach their actions with minimal intervention to avoid interfering with the natural recovery process. This allows different approaches from various agents, both community and professional, with varying levels of specialization. PFA differs from advanced psychological intervention (API) in that PFA provides immediate, basic support and can be administered by those affected themselves, peers, or volunteers with basic training, while API is for more severe cases and requires specialized training.

**Providing PFA knowledge to non-professionals is crucial** as it enables immediate support and intervention, helping reduce the impact of ASRs. New, immediate, focused, safe, and effective PFA approaches are still needed (9, 10).

This Research Topic “*Promoting and exploring the effectiveness of the psychological first aid approach*” aims to explore the effectiveness of implemented PFA protocols in various contexts worldwide.

## Brief review of articles in this Research Topic

Mental distress often follows a stroke and affects recovery. The study by Guo et al. used advanced statistics to examine mental distress trajectories and demographic predictors in stroke patients over four time points. Although the study provided only the methodology and no results, it is relevant to PFA. Understanding potential reactions in advance can help plan preventative PFA interventions to alter negative trajectories and improve prognosis after stroke.

The well-structured study “*Positive mental health and sense of coherence among emergency medical service professionals*” (Mantas-Jiménez et al.) investigates the relationship between Positive Mental Health (PMH) and Sense of Coherence (SOC) among 406 healthcare professionals in the emergency ambulance service in Catalonia, Spain. Main findings reveal that high scores in PMH were significantly associated with greater job satisfaction and a strong SOC. The research identifies Meaningfulness and Comprehensibility, dimensions of SOC, as predictors of higher PMH. Furthermore, the study's findings relate to the principles of PFA and underline the importance of SOC in promoting PFA.

The study “*Pilot evaluation of a Psychological First Aid online training for COVID-19 frontline workers in American Indian/Alaska Native communities*” by O'Keefe et al. evaluated a culturally adapted PFA online training for COVID-19 frontline workers in American Indian/Alaska Native communities. Participants reported high satisfaction and increased PFA knowledge. The training significantly improved positive mental health, social wellbeing, and reduced burnout after 3 months. Despite the lack of a control group, the study is important because it provides a cultural perspective on PFA and highlights the increased attention to emotional wellbeing during the COVID-19 pandemic.

The article “*Assessing the role of sustainability competencies in enhancing psychological first aid effectiveness for disaster responders in Fiji*” examines how trained and untrained disaster responders value sustainability competencies (critical thinking, integrated problem solving, systems thinking, anticipatory normative, collaboration, strategic, and self-awareness), finding that importance varies by age, experience, and training (Nair and Meirmanov). Younger and less experienced responders rate all competencies as very important, while more experienced responders with PFA training particularly emphasize integrated problem-solving, collaboration, and self-awareness.

In conclusion, the articles in this Research Topic illustrate the need for PFA, its accessibility to non-professionals, and the importance of identifying those in need of targeted interventions. Structured PFA training programs improve mental health outcomes. The primary recommendation is to expand PFA training to both non-professionals and professionals across various fields.
